# Uniconazole-induced starch accumulation in the bioenergy crop duckweed (*Landoltia punctata*) II: transcriptome alterations of pathways involved in carbohydrate metabolism and endogenous hormone crosstalk

**DOI:** 10.1186/s13068-015-0245-8

**Published:** 2015-04-11

**Authors:** Yang Liu, Yang Fang, Mengjun Huang, Yanling Jin, Jiaolong Sun, Xiang Tao, Guohua Zhang, Kaize He, Yun Zhao, Hai Zhao

**Affiliations:** Chengdu Institute of Biology, Chinese Academy of Sciences, No.9 Section 4, Renmin Nan Road, 610041 Chengdu, China; University of Chinese Academy of Sciences, No.19A Yuquan Road, 100049 Beijing, China; Key Laboratory of Environmental and Applied Microbiology, Chinese Academy of Sciences, No.9 Section 4, Renmin Nan Road, 610041 Chengdu, China; Environmental Microbiology Key Laboratory of Sichuan Province, No.9 Section 4, Renmin Nan Road, 610041 Chengdu, China; Key Laboratory of Bio-Resources and Eco-Environment, Ministry of Education, College of Life Sciences, Sichuan University, N0.24 South Section 1, Yihuan Road, 610064 Chengdu, China

**Keywords:** Bioethanol, Starch accumulation, Endogenous hormones, Uniconazole, Crosstalk, Pathway

## Abstract

**Background:**

*Landoltia punctata* is a widely distributed duckweed species with great potential to accumulate enormous amounts of starch for bioethanol production. We found that *L. punctata* can accumulate starch rapidly accompanied by alterations in endogenous hormone levels after uniconazole application, but the relationship between endogenous hormones and starch accumulation is still unclear.

**Results:**

After spraying fronds with 800 mg/L uniconazole, *L. punctata* can accumulate starch quickly, with a dry weight starch content of up to 48% after 240 h of growth compared to 15.7% in the control group. Electron microscopy showed that the starch granule content was elevated after uniconazole application. The activities of key enzymes involved in starch synthesis were also significantly increased. Moreover, the expression of regulatory elements of the cytokinin (CK), abscisic acid (ABA) and gibberellin (GA) signaling pathways that are involved in chlorophyll and starch metabolism also changed correspondingly. Importantly, the expression levels of key enzymes involved in starch biosynthesis were up-regulated, while transcript-encoding enzymes involved in starch degradation and other carbohydrate metabolic branches were down-regulated.

**Conclusion:**

The increase of endogenous ABA and CK levels positively promoted the activity of ADP-glucose pyrophosphorylase (AGPase) and chlorophyll content, while the decrease in endogenous GA levels inactivated α-amylase. Thus, the alterations of endogenous hormone levels resulted in starch accumulation due to regulation of the expression of genes involved in the starch metabolism pathway.

**Electronic supplementary material:**

The online version of this article (doi:10.1186/s13068-015-0245-8) contains supplementary material, which is available to authorized users.

## Background

Environmental pollution, global warming, and energy shortages are urgent problems for sustainable development. To gradually decrease our excessive dependence on oil and reduce greenhouse gas emissions, many countries are looking for alternative energy sources. Renewable and clean bioethanol is a promising alternative to oil. However, most feedstocks for bioethanol production are terrestrial crops, such as corn, cassava, and sweet potato, which may compete with food or feed crops for agricultural land and may lead to other environmental problems [1-3]. Therefore, it is necessary to explore novel feedstocks to make the development of the bioethanol industry more sustainable and environmentally friendly.

Duckweed, the smallest and fastest-growing aquatic plant on earth [4], has become a novel potential alternative for bioethanol production in recent years [5]. Duckweed can double its biomass in 16 h to 2 days [6] and hence grows much faster than most other higher plants [7]. The growth rate of duckweed can reach 12.4 g/m^2^/day dry weight in warm regions [8], and its yield has been documented up to 26.50 tons/ha/year dry weight [9]. The dry weight of starch content can reach 75% under ideal growth conditions [10]. Moreover, duckweed can grow on eutrophic wastewater to recover pollution nutrients, and it has been widely applied for wastewater treatment, including industrial wastewater and domestic sewage [11,12]. Importantly, duckweed biomass exhibits good characteristics for bioethanol production due to its relatively high starch and low lignin percentages [13], and it has been successfully converted to bioethanol in recent years [14,15]. Therefore, duckweed could be an ideal candidate for renewable bioenergy sources. Duckweed has great potential to accumulate high starch, and a high starch percentage is the key to energy utilization for duckweed. The starch content of duckweed can be considerably increased by manipulating growing conditions, such as phosphate concentration, nutrient starvation [16], and plant growth regulators [17-19]. Plant growth regulators are common and efficient synthetic compounds that are widely used to regulate plant growth and development [20,21]. To obtain high quality and quantity duckweed for bioethanol utilization, we systematically screened more than 20 plant growth regulators, including auxin, cytokinins (CKs), abscisic acid (ABA), and gibberellins (GAs), to improve the starch and biomass yield of duckweed. Screening results showed that uniconazole can be used as an effective candidate for starch and biomass accumulation of duckweed in Hoagland nutrient solution. Uniconazole (S3307) is a potent and active member of the triazole family that was developed as plant growth retardants [22]. It can enhance plant photosynthetic rates, increase soluble protein and total sugar content, elevate yield components in various crop plants [23,24], and change the endogenous hormone content [25]. However, there have been few in-depth studies into the responsive mechanism of plant growth regulators. There has thus far been little research linking uniconazole with expression changes in hormone biosynthesis enzymes and on the roles of certain hormone variations that cause high starch accumulation. Starch is the major storage form of sugar and energy in plants. The synthesis of starch in plant cells begins with the enzyme ADP-glucose pyrophosphorylase (AGPase), which catalyzes the reaction of glucose-1-phosphate with ATP to form ADP-glucose. The ADP-glucose is then used a substrate by starch synthase (SS) enzymes to build up a starch molecule. Branches in the chain are introduced by starch-branching enzymes (SBEs), which hydrolyse 1, 4-glycosidic bonds, and in their place, create 1, 6 bonds with other glucose units. [26,27].

Next-generation sequencing (NGS) technology is a new development of sequencing technology, and it can provide a novel method to uncover transcriptomics data. It is difficult to research metabolic pathways using conventional biological techniques in non-model plants. However, NGS technologies are not limited to detecting transcripts that correspond to existing genomic sequences, it is particularly attractive for non-model plants with genomic sequences that are yet to be determined [28,29]. This technology has been applied to investigations in some non-model plants and was successfully used to study metabolic pathways in duckweed last year [30]. In the accompanying report, we showed that uniconazole elevated chlorophyll content, enhanced the net photosynthetic rate, and altered the endogenous hormone levels of duckweed (data not shown). However, the relationship between the alteration of endogenous hormone levels and starch accumulation is still unclear. In this study, we constructed a comprehensive transcriptome using NGS technology in combination with physiological and biochemical analyses to investigate the process of starch accumulation mediated by endogenous hormones in *Landoltia punctata*.

## Results

### Impact of uniconazole on starch accumulation of *L. punctata*

*L. punctata* 0202, originally collected from Sichuan, China, is a widely distributed duckweed species with great potential for starch accumulation. In this study, frond samples were collected at 13 time points after treatment with uniconazole for measurement of starch percentages and the activity of enzymes related to starch metabolism.

**Figure 1:**
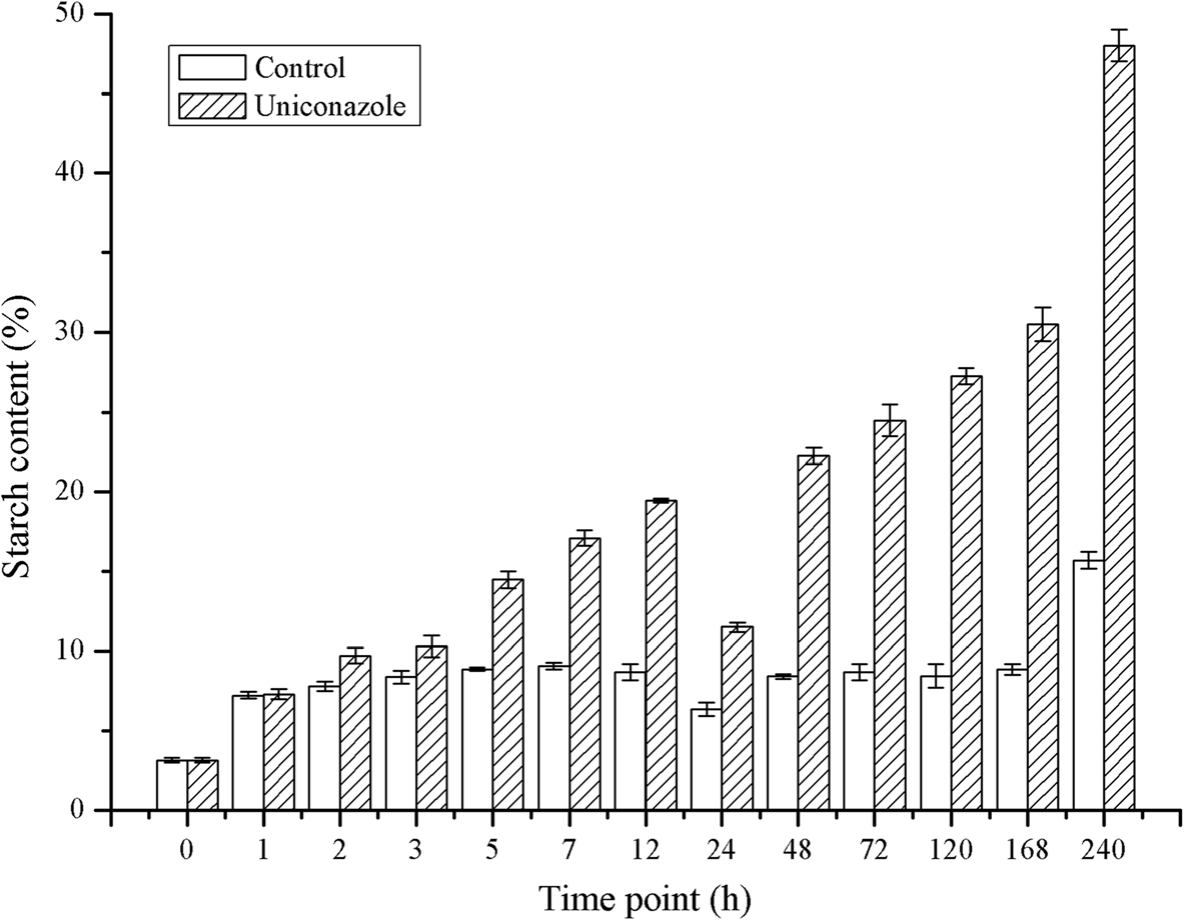
Starch percentage of uniconazole-treated *L. punctata*. Fronds were collected at different time points and used for starch percentage analysis. The starch percentage was calculated basing on dry weight. Each data point represents the mean of triplicate values; error bars indicate the standard deviation.

**Figure 2:**
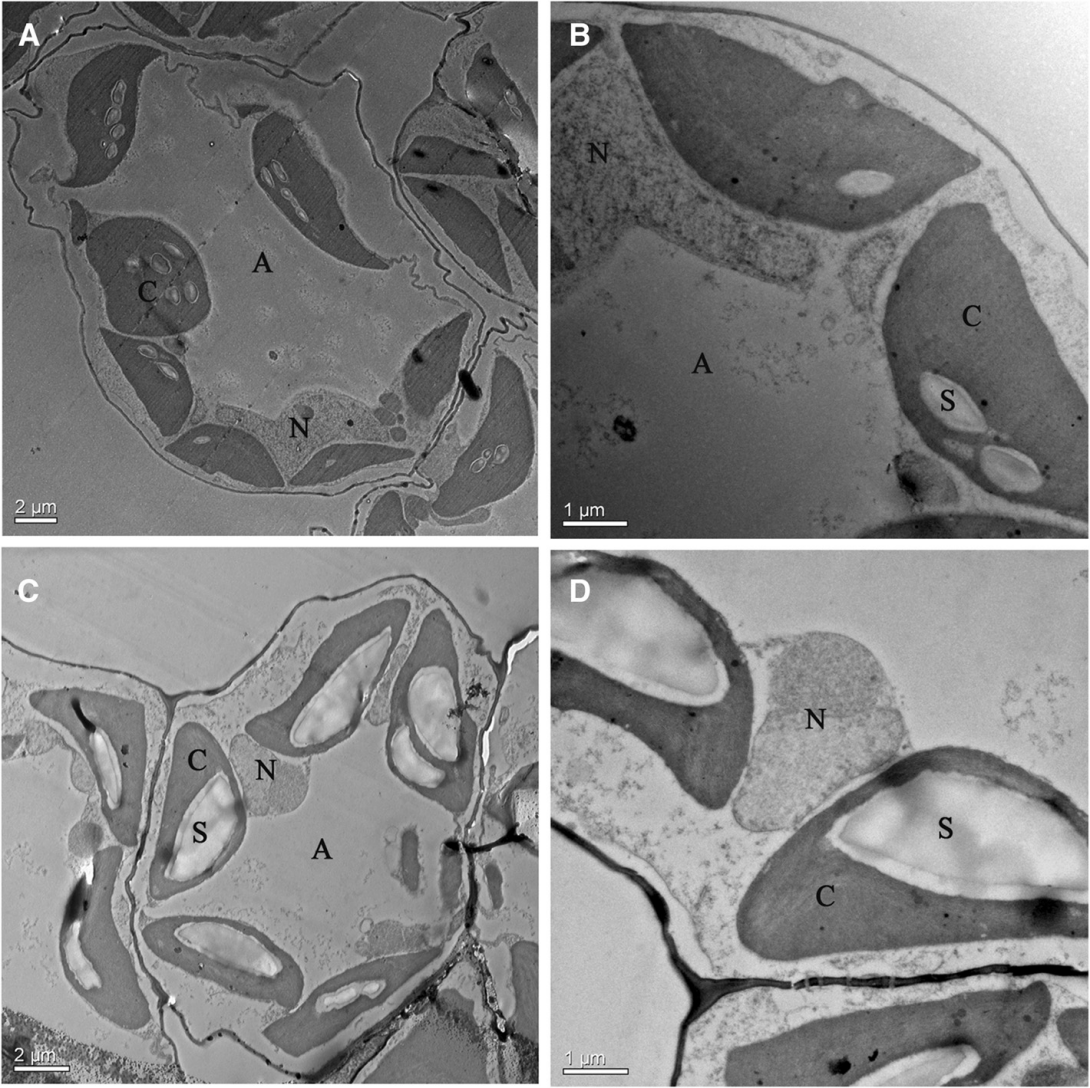
Transmission electron micrographs (TEM) study of *L. punctata*. (**A**) TEM picture of frond cells under lower magnification without treatment, Bars = 2 μm; (**B**) TEM picture of a section of a frond cell under higher magnification without treatment, Bar = 1 μm; (**C**) TEM picture of frond cells under lower magnification treated by uniconazole, Bar = 2 μm; (**D**) TEM picture of a section of a frond cell under higher magnification treated with uniconazole, Bar = 1 μm; Abbreviations are chloroplast (C), starch granule (S), intercellular air space (A), nucleus (N).

As shown in Figure 1, the starch content was 3.16% (DW) at 0 h, but reached 10.31% at 3 h post-treatment. The starch content reached 19.46% (DW) at 12 h and finally reached 48.01% (DW) at 240 h following uniconazole treatment. The starch content in the control fronds over the same time course remained mostly steady, reaching 8.7% (DW) at 12 h and 15.68% (DW) at 240 h post-treatment. Next, frond samples were examined by electron microcopy (Figure 2). The control frond cell contained several chloroplasts with a few small starch granules. In the uniconazole treatment group, several huge starch granules were found in the chloroplasts.

### Effect of uniconazole treatment on the activities of enzymes involved in starch metabolism

**Figure 3:**
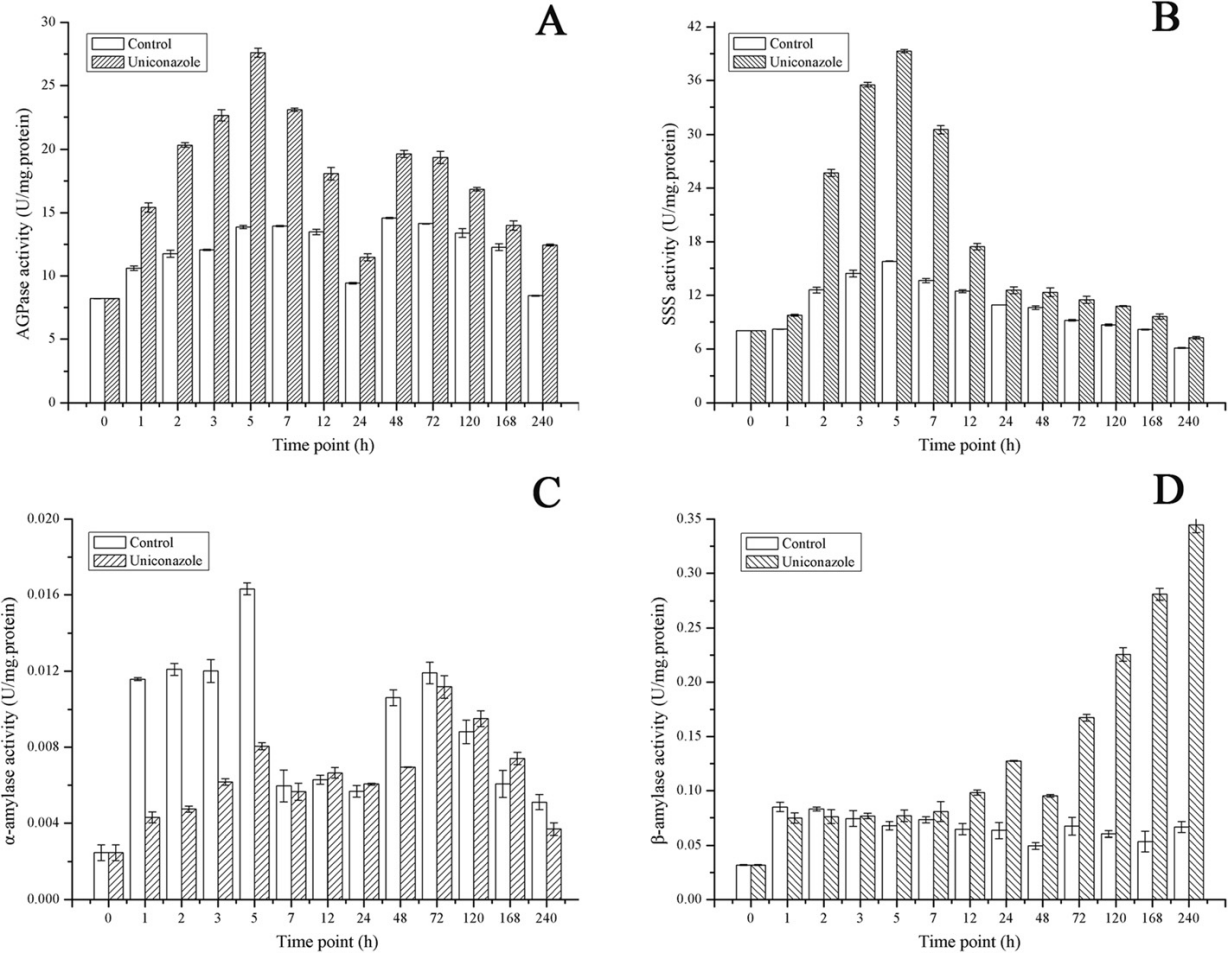
AGPase, SSS, α-amylase, and β-amylase activity. Fronds were collected at different time points and used for starch metabolism-related enzymatic activity assay after uniconazole treatment. (**A**) The activity of ADP-glucose pyrophosphorylase (AGPase); (**B**) The activity of soluble starch synthase (**C**) The activity of α-amylase; (**D**) The activity of β-amylase. All data are presented as the mean of triplicate measurements ± standard deviation.

To gain insight into the rapid accumulation of high starch following uniconazole application, the activity of enzymes involved in starch metabolism was analyzed. The activities of two of the most important key enzymes involved in starch synthesis (starch biosynthesis related enzymes AGPase and soluble starch synthase (SSS)) were measured (Figure 3). The activity of AGPase increased significantly from 8.20 to 27.59 U/mg protein at 5 h. After 24 h, the activity of AGPase increased slightly until 48 h when it reached a nearly stable level, while the activities of AGPase remained mostly constant in the control sample. SSS activity increased from the initial 8.03 to 25.69 U/mg protein at 2 h, and then decreased to 7.24 U/mg protein at 240 h. SSS activity did not change in the control sample.

The activities of starch degradation related enzymes in *L. punctata* were also investigated (Figure 3). The activity of alpha-amylase (α-amylase) was very low and changed very little in both in the control and treatment samples. The activity of α-amylase was 0.0025 U/mg protein at 0 h and reached to 0.037 and 0.0051 U/mg protein in the treated and control samples at 240 h, respectively. However, following treatment, beta-amylase (β-amylase) activity increased gradually from 0.0319 to 0.0747 U/mg protein at 1 h, and finally increased to the 0.3446 U/mg protein at 240 h in the treated sample, with little change in the control sample.

### Sequencing, *de novo* assembly, and functional annotation of the *L. punctata* transcriptome

**Figure 4:**
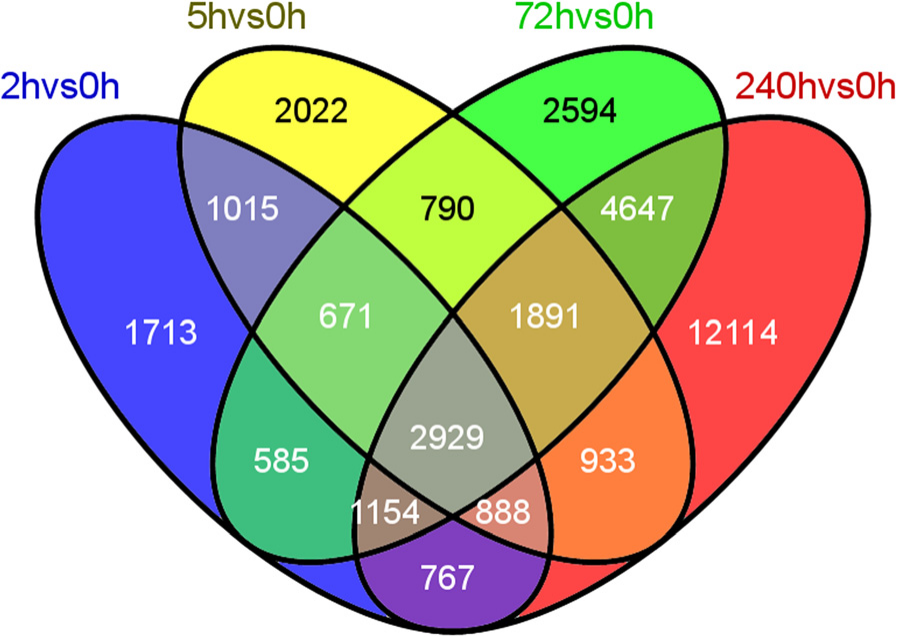
Differential expression between each pair of samples. Venn diagram showing unique and shared genes between time points. Overlapping examinations were performed based on the resulting gene lists from four comparisons by VENNY [74]. Overlap among four groups, 2 *vs* 0 h (blue), 5 *vs* 0 h (yellow), 7 *vs* 0 h (green), and 240 *vs* 0 h (red) are shown.

To investigate the genome-wide expression patterns of uniconazole treated *L. punctata*, samples collected at the 0, 2, 5, 72, and 240 h time points were used for RNA-Seq analysis. Results indicated that most of the contigs were protein-encoding transcripts. For more details on assembly statistics of the *L. punctata* transcriptome, please see the accompanying report (data not shown). To analyze temporal expression patterns of each transcript following uniconazole treatment, all RNA-Seq reads from each *L. punctata* sample were used for mapping analysis. The expression value of each transcript was calculated and normalized according to the RESM-based algorithm. We identified 70,090, 71,268, 71,170, 75,092, and 95,367 transcripts expressed at 0, 2, 5, 72, and 240 h, respectively (Figure 4). According to edgeR [31], compared with 0 h, there were 9,722, 11,139, 15,261, and 25,323 transcripts that were significantly differentially expressed at 2, 5, 72, and 240 h, respectively (Additional file 1: Table S1). Among these four differentially expressed transcripts (DETs: 2 *vs* 0 h, 5 *vs* 0 h, 72 *vs* 0 h, 240 *vs* 0 h), 2,929 transcripts were shared between these four DET sets. Compared with each other in these four DET sets, 5,503 transcripts were shared between 2 *vs* 0 h and 5 *vs* 0 h, 6,281 were shared between 5 *vs* 0 h and 72 *vs* 0 h, 5,339 transcripts were shared between 2 *vs* 0 h and 72 *vs* 0 h, 5,738 transcripts were shared between 2 *vs* 0 h and 240 *vs* 0 h, 6,641 transcripts were shared between 5 *vs* 0 h and 24 *vs* 0 h, and 10,621 were shared between 72 *vs* 0 h and 240 *vs* 0 h.

### Expression analysis of transcript-encoding regulatory proteins and transcription factors involved in the CK, ABA, and GA signaling pathways

**Figure 5:**
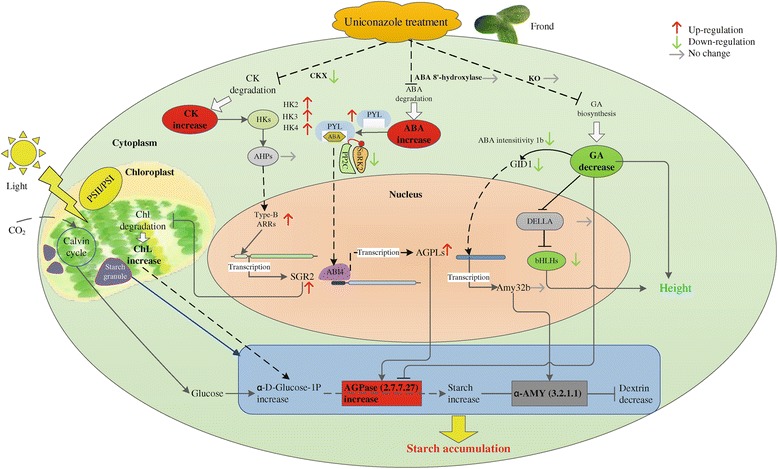
A hypothetical model of cytokinin, abscisic acid, and gibberellin signal pathways related to carbohydrate metabolism. Red indicates up-regulated expression, green down-regulated gene expression, gray means no significant difference was observed, and white means this enzyme was not found in this study. The major signaling pathways are indicated by black lines and arrows. Dotted arrowed lines indicate indirect or unconfirmed connections. Blue arrow indicates enlarged image. Cytokinin is perceived by the cytokinin receptor HKs. Cytokinin binding to HKs activates autophosphorylation (*P*) via AHPs (histidine phosphotransfer proteins) in the cytoplasm. Then type-B *Arabidopsis thaliana* response regulator (type-B ARR) interacts with the promoter of STAY-GREEN2 (SGR2). The family of START proteins (PYLs) act as ABA receptors. ABA combines with intracellular PYL and type 2C protein phosphatase (PP2C) to form an ABA-PYL-PP2C complex. This complex inhibits the activity of PP2C in an ABA-dependent manner and activates SNF1-related protein kinase 2 families (SnRK2s). Abscisic acid insensitive 4 (ABI4) induces ADP-glucose pyrophosphorylase subunit AGPLs (*ApL3*) gene expression. The main components of the GA signal pathway include GA receptor (GID1) and DELLA growth inhibitors. The GA-GID1-DELLA complex stimulates the degradation of DELLAs to regulate plant growth. GID1 regulated the transcription of amylase by a number of transcriptional regulatory.

The transcripts of regulatory proteins and transcription factors involved in the CK, ABA, and GA signaling pathways changed significantly in response to uniconazole treatment (Figure 5). Cytokinins are degraded by cytokinin oxidase/dehydrogenase (CKXs) which is thought to play a key role in controlling cytokinin levels in plants. The expression of CKX was down-regulated from 11.97 fragments per kilobase of transcripts per million mapped fragments (FPKM) at 0 h to 4.22 FPKM at 240 h. In the signaling pathway of cytokinin, the cytokinin receptors histidine kinases (HKs) were up-regulated. For example, HK3 expression increased from 27.22 to 49.06 FPKM at 72 h (Additional file 2: Table S2). Their downstream elements histidine phosphotransfer proteins (AHPs) carry conserved amino acids required for phosphotransfer via a conserved histidine residue. There was no significant change in expression of AHPs. Type-B *Arabidopsis thaliana* response regulator (type-B ARR) interacts with the promoter of STAY-GREEN2 (SGR2) and interrupts transcription of the chlorophyll degradation pathway. SGR expression was up-regulated significantly from an initial value of 8.46 to 130.77 FPKM.

The core ABA signaling components have been well described in recent years. The family of START proteins (PYLs) act as ABA receptors, and 13 of 14 members of the Arabidopsis PYL family have been identified. PYL1 and PYL8 were identified in duckweed, and the expression levels of PYL1 increased from 36.88 FPKM at 0 h to 45.27 FPKM at 240 h. The expression of the PP2C negative regulator (comp31119_c0_seq1) decreased from 308.55 FPKM at 0 h to 122.48 FPKM at 240 h. There were no significant changes observed for the expression of transcript-encoding abscisic acid insensitive 4 (ABI4) (comp31717_c0_seq1). The expression was 0.14, 0.22, 0.05, 0.09 and 0.14 FPKM, respectively. The expression of two identical large subunits of AGPase (AGP-LS comp37255_c0_seq1) was up-regulated from the initial value of 122.13 to 192.48 FPKM at 240 h (Additional file 2: Table S2).

In the GAs signal pathway, transcriptomics data showed that the expressions of GA receptor GA insensitive dwarf 1 (GID1 comp41567_c0_seq1) were down-regulated from 44.03 to 23.93, 28.28, and 33.5 FPKM at different time points. The expressions of transcript-encoding DELLA proteins (comp33138_c1_seq1) were down-regulated from 6.3 to 4.55, 3.05, 3.61, and 2.12 FPKM, respectively. Moreover, the expression levels of transcript-encoding α-amylase were down-regulated from the initial value of 8.03 to 6 FPKM.

### Expression analysis of transcript-encoding key enzymes involved in starch accumulation

**Figure 6:**
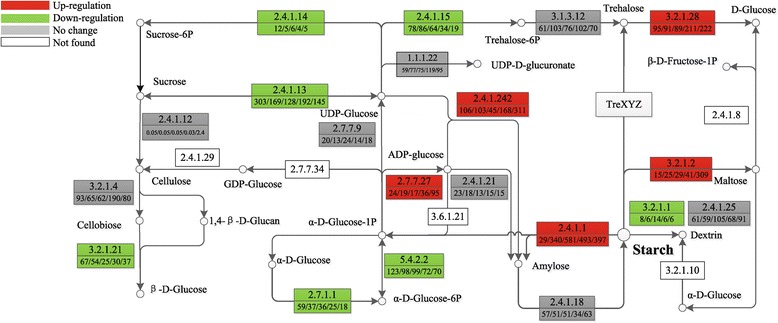
Expression patterns of carbohydrate metabolism-related transcripts. Expression variations of some carbon metabolism-related transcripts are displayed in the simplified starch and sucrose metabolism pathway. Red boxes indicate the up-regulated enzymes involved in carbohydrate metabolism, green boxes indicate the down-regulated enzymes, gray boxes mean no significant difference was observed, and white boxes mean this enzyme was not found in this study. The numbers in the upper half of the boxes correspond to the EC numbers and the numbers in the lower half, separated by slashes, correspond to the expression levels of these enzymes shown in FPKM at 0, 2, 5, 72, and 240 h, respectively. 1.1.1.22: UDP-glucose 6-dehydrogenase; 2.4.1.1: glycogen phosphorylase; 2.4.1.13: sucrose synthase; 2.4.1.14: sucrose phosphate synthase; 2.4.1.21: soluble starch synthase; 2.4.1.15: trehalose-6-phosphate synthase; 2.4.1.18: starch-branching enzyme; 2.4.1.12: cellulose synthase; 2.4.1.242: granule bound starch synthase; 2.7.7.27: ADP-glucose pyrophosphorylase; 2.7.7.9: UDP-glucose pyrophosphorylase; 2.7.1.1: hexokinase; 3.2.1.1: alpha-amylase; 3.2.1.2: beta-amylase; 3.1.3.12: trehalose 6-phosphate phosphatase; 3.2.1.4: endoglucanase; 3.2.1.28: trehalase; 5.4.2.2: phosphoglucomutase.

Starch is the major storage carbohydrate in plants. To investigate the mechanisms by which uniconazole treatment resulted in starch accumulation, the expression patterns of transcript-encoding key enzymes were analyzed (Figure 6). Gene expression profiling results showed that the expression of transcript-encoding AGPase were up-regulated from 24 to 95 FPKM at 240 h (comp37852_c0_seq1). Transcript-encoding granule-bound starch synthase (GBSS) exhibited an expression level of 106 FPKM at 0 h and increased to 311 FPKM at 240 h (comp31254_c0_seq1). There were no significant changes observed for the expression of transcript-encoding SSS and SBE (Additional file 3: Table S3).

Transcript-encoding enzymes involved in starch degradation and other carbohydrate metabolic branches were also analyzed. The expression level of transcript-encoding trehalose-6-phosphate synthase (EC: 2.4.1.15; TPS), which catalyze the biosynthesis of trehalose using UDPGlucose as substrate, was down-regulated from 78.1 to 18.7 FPKM (comp36386_c1_seq6) at 240 h after uniconazole treatment. Carbohydrate metabolic branches that compete with the synthesis of starch were also measured, including hexokinase (EC: 2.7.1.1), beta-glucosidase (EC: 3.2.1.21), phosphoglucomutase (EC: 5.4.2.2), sucrose-phosphate synthase (EC: 2.4.1.14), and sucrose synthase (SuSy EC: 2.4.1.13). Specifically, hexokinase (comp24929_c1_seq2) was significantly down-regulated from 59 to 18 FPKM at 240 h. No significant increase was observed for the expression of transcript-encoding α-amylase. However, transcript-encoding β-amylase exhibited an expression level of 15 FPKM at 0 h and increased to 309 FPKM at 240 h (comp16912_c0_seq1).

## Discussion

### The relationship between endogenous hormones induced by uniconazole and starch accumulation in *L. punctata*

Numerous studies have investigated different types of plant growth regulators that regulate growth and development in plants. Some articles focus their investigations on certain stress responses mediated by one or two types of plant hormones [32-34]. These studies often analyze phenotypic, biochemical, and physiological data. Some studies research the function of regulatory elements on hormones of signaling pathways [35,36]. Other studies investigate the relationship between different types of hormones [37-39]. Regulatory proteins are the main focus in these articles, which utilize molecular biology techniques with little to no focus on metabolic pathways. Additionally, some articles used transcriptome analyses to study the response of plants treated with hormones [40-42]; however, these articles emphasized the up- or down-regulation of genes or discovery of new genes, but did not consider metabolic pathways. Li [43] used RNA sequencing technology to understand the mechanisms of parthenocarpy and predicted 14 genes as putative parthenocarpic genes. The transcription analyses of these candidate genes revealed that auxin, cytokinin, and gibberellin crosstalk at the transcriptional level during parthenocarpic fruit set, but the metabolic pathways of these hormones were not mentioned. In this study, we analyzed metabolic pathways using NGS technology; this data, combined with physiological and biochemical analyses and crosstalk of different plant hormones, allowed us to elucidate the process of starch accumulation in *L. punctata*.

The alteration of the endogenous CK, ABA, and GAs co-regulates starch metabolism in *L. punctata*. The endogenous hormone content changed dramatically following uniconazole application. ABA content increased from 61.47 to 166.53 ng/g (FW), ZR increased from 7.73 to 11.87 ng/g (FW), and the level of GA_1+3_ decreased from 9.25 to 5.57 ng/g (FW) following treatment (accompanying report). CKs elevated the chlorophyll content by controlling regulatory proteins involved in the chlorophyll biosynthesis signaling pathway. CKs are a class of plant growth substances that promote cell division, chloroplast synthesis, and amyloplast formation [44,45]. Furthermore, the increase of CKs plays an important role in regulating grain filling pattern and consequently elevated starch accumulation [46]. In the CK mediated chlorophyll synthesis signaling pathway [47,48], CKs are degraded by CKXs. The transcriptomics data suggested that HKs and SGR were up-regulated. The expression of SGR was up-regulated significantly from the initial value of 8.46 to 130.77 FPKM. The interaction of type-B ARR with SGR2 was assayed to determine whether the cytokinin signaling pathway interacted with a key step in chlorophyll degradation within the chloroplast [49]. The increase in SGR can suppress the degradation of chlorophyll, thereby improving chlorophyll content. Furthermore, the expression pattern of the regulatory proteins described above coincided with the increase in chlorophyll content. The chlorophyll a content increased from the initial value of 0.998 to 1.239 mg/g (FW), and chlorophyll b increased from the initial value of 0.426 to 0.488 mg/g (FW). The chlorophyll a and chlorophyll b content increased by 25.6% and 27%, respectively, compared to the control sample. Importantly, the improvement of the net photosynthetic rate was consistent with the increase of starch and biomass accumulation. The net photosynthetic rate increased from the initial value of 8.83 μmol CO_2_/m^2^/s to 22.05 and 25.6 μmol CO_2_/m^2^/s in the control and treatment groups, respectively (accompanying report). Thus, the improvement of chlorophyll content and net photosynthetic rate may lead to starch and biomass accumulation in *L. punctata.*

ABA up-regulated the expression of AGPase large subunit gene transcription by controlling the expression of regulatory elements of the ABA signal pathway. Studies have shown that ABA can up-regulate AGPase gene transcription in rice suspension cells [50] and suppress the expression of gene-encoding amylases and proteases [51]. Reports also indicated that the rates of starch accumulation are positively correlated with ABA levels in wheat grains. As shown in Figure 5, the expression of transcript-encoding ABA receptors of PYLs was up-regulated from 36.88 to 45.27 FPKM at 240 h. ABA combines with intracellular PYL and negative regulator PP2C (type 2C protein phosphatase) to form an ABA-PYL-PP2C complex. ABI4 induces ADP-glucose pyrophosphorylase subunit (*ApL3*) gene expression [52-55]. Moreover, the expression of the AGPase large subunit gene (*ApL3*) also increased. Importantly, the up-regulated expression of the AGPase large subunit gene strongly supported the improved activity of AGPase, and the increased activity of AGPase promoted starch accumulation in duckweed.

GAs suppressed the expression of the amylase gene by controlling expression of regulatory factors. A study of GA regulating the growth and carbohydrate metabolism of potatoes showed that GA_3_ could substantially reduce the activity of AGPase in the growing tubers of potatoes [56]; therefore, uniconazole treatment might eliminate the obstacle by blocking GA synthesis and enhancing starch accumulation. GAs can induce or activate α-amylase and other hydrolases, which is not conducive to the synthesis and accumulation of starch [57,58]. Moreover, reports showed that during grain filling, the ratio of endogenous GAs and ABA changes greatly in rice. The ABA content was significantly increased and GA content dramatically decreased, which enhanced the remobilization of prestored carbon to the grains and accelerated the grain filling rate [59]. In this study, the expression of α-amylase was down-regulated from the initial 8.03 to 6 FPKM. The decrease of GA levels suppressed α-amylase expression, and the reduced activities of α-amylase prevented starch degradation in duckweed. These findings also support starch accumulation.

The improvement of starch accumulation following uniconazole treatment was closely associated with the elevated level of endogenous ABA and CK and reduced GA content in duckweed. In this study, high levels of CKs accelerated the biosynthesis of chlorophyll and the net photosynthetic rate, the increased ABA content promoted the activity of AGPase, and the low levels of GAs inactivated amylase. Overall, the alterations in endogenous hormone levels following uniconazole treatment improved starch accumulation in duckweed by influencing the related enzymes involved in carbohydrate metabolism and processes.

### Starch accumulation of *L. punctata* under uniconazole treatment

The rapid starch accumulation in fronds of *L. punctata* after uniconazole treatment displayed some similarities to grain filling which is a major process of starch biosynthesis and accumulation in seeds. For instance, both processes are rapid and show similar alteration of endogenous hormone levels such as a decrease in GAs and an increase in ABA. In addition, some key enzymes (AGPase and SSS) involved in starch biosynthesis are regulated in a similar way in both processes.

In this study, the transcriptomics analyses, enzymatic assays, and starch percentages were integrated to uncover the process of rapid accumulation of high starch after uniconazole application. The data from three lines of evidence were analyzed and compared. Investigation of starch composition showed that the starch content and biomass yield in *L. punctata* accumulated rapidly. After culturing for 240 h, the starch content reached 48% in the treated samples and 15.7% in the control samples from an initial yield of 3.2% (Figure 1). The biomass of treated samples (dry weight) improved 10% over the control samples (data not shown). As a result, the total starch that accumulated in the treated samples was 3.4 times higher than that in the control samples. Meanwhile, the enzyme activities involved in starch synthesis, such as AGPase and SSS, were improved dramatically by uniconazole treatment (Figure 3). The activity of AGPase increased significantly from 8.20 to 27.59 U/mg protein, representing a 3.4-fold increase. Importantly, the expression patterns of transcript-encoding key enzymes involved in starch biosynthesis and degradation further supported the physiological and biochemical results described above. Transcriptome analysis showed that the expression of GBSS transcripts were up-regulated significantly.

Starch phosphorylation and glucan hydrolysis are two necessary steps in the degradation process. Glucan water dikinase (GWD) and phosphoglucan water dikinase (PWD) are responsible for starch phosphorylation. Β-amylases catalyze the hydrolysis of a-1, 4-glycosidic linkages and release maltose from the exposed nonreducing ends of glucan chains. Α-amylases hydrolyze α-1, 4 linkages within polymers exposed on the surface or in channels within granules, releasing soluble glucans that are the substrate for further degradation [60,61]. In this study, there no significant changes were observed for the expression of transcript-encoding GWD. The expression level of GWD (comp38348_c1_seq1) was 40.24, 39.54, 63.45, 39.05, and 47.73 FPKM in different time points, respectively. The starch degradation enzyme activities of α-amylase changed little between the control and treated samples. Though the expression of β-amylase was increased in a manner contradictory with starch accumulation, physiological data showed that the activity of β-amylase was too low to compare with the improved enzyme activity of starch biosynthesis. The expression of enzymes involved in competitive starch metabolic branches, including hexokinase sucrose-phosphate synthase, phosphoglucomutase (EC: 5.4.2.2) and others were also down-regulated (Figure 6). Coupled with the up-regulation of starch biosynthesis related key enzyme-encoding transcripts, the down-regulation of transcripts finally redirected alpha-D-glucose-1P and UDP-glucose to the starch biosynthesis branch.

In this study, up-regulation of key enzymes in starch biosynthesis, in combination with down-regulation of transcripts of key enzymes related to starch degradation and other carbohydrate metabolic branches that compete with the synthesis of starch, eventually led to the accumulation of starch in *L. punctata*.

Uniconazole has a similar chemical structure to paclobutrazol. It reduces plant growth more than paclobutrazol when applied as a soil drench in equal amounts. On average, the amount of paclobutrazol required is four to ten times that of uniconazole, to obtain a similar effect on plant size [62]. Early research indicated that uniconazole can be very persistent in retarding plant growth without causing phytotoxicity [63]. Half-lives of paclobutrazol and uniconazole in water were 24.4 and 5.2 days, respectively. Uniconazole-p is non-toxic to birds, bees, and earthworms, but slightly toxic to fish and aquatic invertebrates. Therefore, it can be applied to high starch accumulation of duckweed in large-scale cultivation.

## Conclusions

In this study, high starch accumulation in *L. punctata* 0202 was achieved after uniconazole application in a nutrient-rich environment. The process of starch accumulation was investigated at physiological, biochemical, and transcriptome levels. The increase in endogenous ABA and CK levels further enhanced the activity of AGPase and chlorophyll biosynthesis, while decreased endogenous GA levels significantly correlated with the inactivation of α-amylase. Moreover, uniconazole increased the levels of substrates of starch synthesis and regulated transcriptional expression of enzymes by changing the biosynthesis of endogenous hormones, resulting in starch accumulation in duckweed. Because of the complex interaction among different hormones, the alteration of endogenous hormone levels can provide further insight into the relationship of endogenous hormones to starch accumulation. In this study, an operable process for high starch accumulation in duckweed was developed, paving the way for large-scale treatment of wastewater and the application of duckweed to bioenergy.

## Materials and methods

### Duckweed cultivation and uniconazole treatments

*L. punctata* 0202 was originally collected from Sichuan province, China. It was cultivated in standard 1/6Hoagland E+ solution (Total *N* = 58.3 mg/L, *P* = 25.8 mg/L) [64] culture for 3 days under a 16/8 h day/night photoperiod, with a light intensity of 130 μmol/m^2^/s and a temperature of 25°C/15°C at day/night. Then, 6 g of fronds were transferred into 1,000 mL 1/6 Hoagland E+ culture plastic containers (23 × 14 × 4.5 cm) for further cultivation over a period of 10 days. Uniconazole powder was produced in Japan and purchased from Aoke Biotech Corp (Beijing, China). The concentration of uniconazole used in this study was 800 mg · L^−1^. To investigate the effect of uniconazole treatment on *L. punctata*, a 5-mL solution of 800 mg · L^−1^ uniconazole was sprayed evenly on the surface of fronds. Controls were sprayed with 5 mL water containing 10% methanol. The experiments were carried out with three replicates. Thirteen different time points, including 0, 1, 2, 3, 5, 7, 12, 24, 48, 72, 120, 168, and 240 h after fronds were cultured in solution and were chosen for composition and enzymatic activity assays. For each time point, fronds were collected from three culture plastic containers. Samples collected at 0, 2, 5, 72, and 240 h were frozen in liquid nitrogen immediately for the RNA-Seq study.

### Material composition

The starch content was described as glucose content in total sugar by HPLC (Thermo 2795, Thermo Corp, Waltham, USA)-ELSD (All-Tech ELSD 2000, All-tech, Corp, Nicholasville, USA) using the following method. The starch content was determined using the total sugar content (starch content = glucose content × 0.909). Dry duckweed powder was hydrolyzed with 1.2 M HCl in a boiling water bath. After adjusting the pH to 7 with 10 M NaOH, PbAc was added to precipitate protein. After the solution was measured, filtered, and treated with a C18 extraction column, the hydrolyzate was analyzed by HPLC (Thermo 2795, Thermo Corp.) with an Evaporative Lightscattering Detector (All-Tech ELSD 2000, All-tech., Corp.) [65].

### Microscopic analysis of fronds

Fronds in the uniconazole treatment group and control group were fixed, embedded, and dehydrated as described [66,67]. Samples were fixed in 5% glutaraldehyde in 0.1 M PBS (pH 7.4) containing 2% Suc in a 2 mL tube at 4°C overnight followed by 3 h at room temperature. Samples were rinsed with 0.1 M PBS (pH 7.4) and postfixed in buffered 1% osmium tetroxide at 4°C overnight, followed by dehydration in a graded series of acetone washes. The dehydrated samples were then embedded in epon resin. The 1 mm-thick sections were picked up on a glass slide, stained with methylene blue, and scoped with a light microscope. Ultrathin sections were cut with an ultramicrotome (Leica EM UC6, Wetzlar, Germany) and observed with transmission electron microscopy (TEM; Tecnai G^2^ F20S-Twin, FEI, Hillsboro, USA) at 200 kV after staining with uranyl acetate and lead citrate.

### Carbohydrate metabolism enzyme activity assay

To investigate enzyme activity, 1 g fresh weight duckweed was homogenized with a ceramic pestle in an ice-cold mortar in 5 mL of 50 mmol/L HEPES-NaOH (pH = 7.6), 5 mmol/L DL-Dithiothreitol, 8 mmol/L MgCl_2_, 2 mmol/L EDTA, 2% (*w*/*v*) polyvinylpyrrolidone-40, and 12.5% (*w*/*v*) glycerol. The homogenate was centrifuged at 10,000 × *g* for 5 min. The supernatant extract was used as a crude enzyme solution stored at −20°C. All procedures were carried out at 0 to 4°C. The activities of α-amylase (1, 4, d-glucan glucanohydrolase) and β-amylase (1, 4, d-glucan maltohydrolase) were estimated following the method of Tarrago and Nicolas [68,69]. The enzymatic activities of SSS and AGPase were assayed according to Nakamura *et al.* [69].

### RNA extraction and cDNA fragment library construction

Five *L. punctata* samples were collected at the 0, 2, 5, 72, and 240 h time points after treatment with uniconazole. For each sample, total RNA was extracted from 200 mg fronds using the OMEGATM Plant DNA/RNA kit (OMEGA, Norcross, USA) and genomic DNA was digested by DNase I (Fermentas, Waltham, USA) according to the manufacturer’s instructions. RNA concentration, OD260/280, OD260/230, 28S/18S and RNA integrity number (RIN) were measured with the Agilent 2100 Bioanalyzer or NanoDrop (Agilent, Santa Clara, USA). Qualified total RNA extracted from each sample was submitted to the Beijing Genomics Institute (BGI)-Shenzhen, Shenzhen, China, (http://www.genomics.cn) for RNA sequencing by Illumina HiSeq 2000 (Illumina, San Diego, USA). cDNA fragment libraries were constructed according to the manufacturer’s instructions using the TruSeq RNA Sample Prep kit. Library quality control analysis was performed using the Agilent 2100 Bio-analyzer.

### RNA sequencing and paired-end reads assembly

The validated 200 bp fragment cDNA libraries were submitted to the Illumina HiSeq 2000 platform for paired-end (PE) RNA sequencing. PE read sequencing quality was assessed by fastqc (http://www.bioinformatics.bbsrc.ac.uk/projects/fastqc/) and then *de novo* assembled using Trinity (v2012-06-08) [70] under default parameter choices. All PE reads were used to align back to these assembled sequences using the Bowtie2 (v2.0.0-beta5) program [71]. Accordingly, the read align rate was calculated. Length distribution analysis was performed with Perl scripts (Additional file 4) to calculate the N50 number, average length, and max length. The best candidate open reading frame (ORF) was predicted using Perl scripts in the Trinity package (v2012-06-08) [70].

### Functional annotation and cluster

All contigs assembled by Trinity (v2012-06-08) [70] were submitted to Blast2GO [72,73] for functional annotation. A BLASTX similarity search was performed against the NR database (http://www.ncbi.nlm.nih.gov/) by Blast2GO with a threshold of *E* value <10^3^. Enzyme codes were extracted, and Kyoto Encyclopedia of Genes and Genomes (KEGG) pathways were retrieved from the KEGG web server (http://www.genome.jp/kegg/).

### Expression pattern analysis

To analyze the express levels of each transcript at different time points following uniconazole treatment, all PE reads for each sample were used for mapping analysis with Perl scripts in the Trinity package (v2012-06-08) [70] under default parameter choices. The expression value of each transcript was calculated and normalized according to the RESM-based algorithm using the Perl scripts in the Trinity (v2012-06-08) package to obtain FPKM values. *P* values and log_2_ fold change (log_2_FC) were calculated, and significantly DETs between each sample set were identified with *P* value ≤0.05 and log_2_FC ≥1. Hypergeometric tests based on the KEGG annotation were performed for each DET group identified between each sample set using R scripts (Additional file 4) to extract the enriched KEGG pathway. Additionally, we depicted differences and commonalities in the number of DEGs using the VENNY under default parameter choices (http://bioinfogp.cnb.csic.es/tools/venny/index.html) [74].

### Calculations and statistics

Each data point represents the results of three sample experiments; the results are provided as means ± standard error in the figures.

## Additional files

Additional file 1: Table S1. Sequence annotations of *L. punctata* transcripts and the gene expression profiling of five samples. Additional file 2: Table S2. Expression levels of some regulatory proteins and transcription factors involved in CK, ABA, and GA signaling pathways. Additional file 3: Table S3. Expression levels of some carbon metabolism related genes. Additional file 4:
**Common scripts.** The common scripts including Perl scripts and R scripts for assembly statistic and pathway enrich.

## Abbreviations

ABA: abscisic acid
ABI4: abscisic acid insensitive 4
AHPs: histidine phosphotransfer proteins
CKs: cytokinins
DET: differentially expressed transcript
DW: dry weight
EC: enzyme codes
FPKM: fragments per kilobase of transcripts per million mapped fragments
FW: fresh weight
GAs: gibberellins
HKs: histidine kinases
KEGG: Kyoto Encyclopedia of Genes and Genomes
log2FC: log2 fold change
NGS: next-generation sequencing
PE: paired-end
PP2C: type 2C protein phosphatase
PYLs: family of START proteins
SGR2: STAY-GREEN2 protein
type-B ARR: type-B *Arabidopsis thaliana* response regulator

## References

[1] Ge L, Wang P, Mou H (2011). Study on saccharification techniques of seaweed wastes for the transformation of ethanol. Renew Energ.

[2] Crutzen PJ, Mosier AR, Smith KA, Winiwarter W (2008). N2O release from agro-biofuel production negates global warming reduction by replacing fossil fuels. Atmos Chem Phys.

[3] Searchinger T, Heimlich R, Houghton RA, Dong F, Elobeid A, Fabiosa J (2008). Use of US croplands for biofuels increases greenhouse gases through emissions from land-use change. Science.

[4] Landolt E (1986). Biosystematic investigations in the family of duckweed (Lemnaceae).

[5] Ge X, Zhang N, Phillips GC, Xu J (2012). Growing Lemna minor in agricultural wastewater and converting the duckweed biomass to ethanol. Bioresour Technol..

[6] Leng R, Stambolie J, Bell R (1995). Duckweed-a potential high-protein feed resource for domestic animals and fish. Livest Res Rural Dev.

[7] Hillman WS, Culley DD (1978). The uses of duckweed. Am Sci..

[8] Xu J, Cui W, Cheng JJ, Stomp A-M (2011). Production of high-starch duckweed and its conversion to bioethanol. Biosyst Eng.

[9] Zhao Y, Fang Y, Jin Y, Huang J, Bao S, Fu T (2014). Potential of duckweed in the conversion of wastewater nutrients to valuable biomass: a pilot-scale comparison with water hyacinth. Bioresour Technol..

[10] Reid M, Bieleski R (1970). Response of Spirodela oligorrhiza to phosphorus deficiency. Plant Physiol.

[11] Bayrakci AG, Kocar G (2014). Second-generation bioethanol production from water hyacinth and duckweed in Izmir: a case study. Renew Sust Energ Rev..

[12] El-Shafai SA, El-Gohary FA, Nasr FA, Peter Van Der Steen N, Gijzen HJ (2007). Nutrient recovery from domestic wastewater using a UASB-duckweed ponds system. Bioresour Technol.

[13] Blazey EB, McClure JW (1968). The distribution and taxonomic significance of lignin in the Lemnaceae. Amer J Bot.

[14] Cui W, Xu J, Cheng J, Stomp A (2011). Starch accumulation in duckweed for bioethanol production. Biol Eng..

[15] Chen Q, Jin Y, Zhang G, Fang Y, Xiao Y, Zhao H (2012). Improving production of bioethanol from Duckweed (Landoltia punctata) by pectinase pretreatment. Energies.

[16] Xiao Y, Fang Y, Jin Y, Zhang G, Zhao H (2013). Culturing duckweed in the field for starch accumulation. Ind Crop Prod..

[17] McLAREN JS, Smith H (1976). The effect of abscisic acid on growth, photosynthetic rate and carbohydrate metabolism in Lemna minor L. New Phytol.

[18] McCombs P, Ralph R (1972). Protein, nucleic acid and starch metabolism in the duckweed *Spirodela oligorrhiza* treated with cytokinins. Biochem J..

[19] Wang W, Messing J (2012). Analysis of ADP-glucose pyrophosphorylase expression during turion formation induced by abscisic acid in Spirodela polyrhiza (greater duckweed). BMC Plant Biol.

[20] Pavlista AD (2011). Growth regulators increased yield of Atlantic potato. Am J Potato Res.

[21] Fletcher RA, Hofstra G (1990). Improvement of uniconazole-induced protection in wheat seedlings. J Plant Growth Regul.

[22] Fletcher R, Hofstra G, Gao JG (1986). Comparative fungitoxic and plant growth regulating properties of triazole derivatives. Plant Cell Physiol.

[23] Zhou W, Ye Q (1996). Physiological and yield effects of uniconazole on winter rape (Brassica napus L.). J Plant Growth Regul..

[24] Wan-zhuo G, Zheng-yi Z, Weng-yu Y, Wen-zhu L (2007). Effect of uniconazloe for dry seed treatment on morphological characteristics and yield of soybean. Soybean Science.

[25] Zhang M, Duan L, Tian X, He Z, Li J, Wang B (2007). Uniconazole-induced tolerance of soybean to water deficit stress in relation to changes in photosynthesis, hormones and antioxidant system. J Plant Physiol.

[26] Smith AM (2001). The biosynthesis of starch granules. Biomacromolecules.

[27] Martin C, Smith AM (1995). Starch biosynthesis. Plant Cell.

[28] Wu Y, Wei W, Pang X, Wang X, Zhang H, Dong B, Xing Y, Li X, Wang M (2014). Comparative transcriptome profiling of a desert evergreen shrub, Ammopiptanthus mongolicus, in response to drought and cold stresses. BMC Genomics.

[29] Wang X, Zhou G, Xu X, Geng R, Zhou J, Yang Y (2014). Transcriptome profile analysis of adipose tissues from fat and short-tailed sheep. Gene.

[30] Tao X, Fang Y, Xiao Y, Jin YL, Ma XR, Zhao Y (2013). Comparative transcriptome analysis to investigate the high starch accumulation of duckweed (Landoltia punctata) under nutrient starvation. Biotechnol Biofuels.

[31] Robinson MD, McCarthy DJ, Smyth GK (2010). edgeR: a Bioconductor package for differential expression analysis of digital gene expression data. Bioinformatics.

[32] Guan C, Wang X, Feng J, Hong S, Liang Y, Ren B (2014). Cytokinin antagonizes abscisic acid-mediated inhibition of cotyledon greening by promoting the degradation of abscisic acid insensitive5 protein in Arabidopsis. Plant Physiol.

[33] Ramireddy E, Chang L, Schmulling T (2014). Cytokinin as a mediator for regulating root system architecture in response to environmental cues. Plant Signal Behav..

[34] Reguera M, Peleg Z, Abdel-Tawab YM, Tumimbang EB, Delatorre CA, Blumwald E (2013). Stress-induced Cytokinin synthesis increases drought tolerance through the coordinated regulation of carbon and nitrogen assimilation in rice. Plant Physiol.

[35] Bastias A, Yanez M, Osorio S, Arbona V, Gomez-Cadenas A, Fernie AR (2014). The transcription factor AREB1 regulates primary metabolic pathways in tomato fruits. J Exp Bot.

[36] Kim HJ, Chiang Y-H, Kieber JJ, Schaller GE (2013). SCFKMD controls cytokinin signaling by regulating the degradation of type-B response regulators. Proc Natl Acad Sci U S A.

[37] Yang D-L, Yao J, Mei C-S, Tong X-H, Zeng L-J, Li Q (2012). Plant hormone jasmonate prioritizes defense over growth by interfering with gibberellin signaling cascade. Proc Natl Acad Sci U S A.

[38] Giulia E, Alessandro B, Mariano D, Andrea B, Benedetto R, Angelo R (2013). Early induction of apple fruitlet abscission is characterized by an increase of both isoprene emission and abscisic acid content. Plant Physiol.

[39] Wang Y, Li L, Ye T, Zhao S, Liu Z, Feng Y-Q (2011). Cytokinin antagonizes ABA suppression to seed germination of Arabidopsis by downregulating ABI5 expression. Plant J.

[40] Wang Y, Tao X, Tang X-M, Xiao L, Sun J-l, Yan X-F, Li D, Deng H-Y, Ma X-R (2013). Comparative transcriptome analysis of tomato (Solanum lycopersicum) in response to exogenous abscisic acid. BMC Genomics.

[41] Chandrasekaran U, Xu W, Liu A: Transcriptome profiling identifies ABA mediated regulatory changes towards storage filling in developing seeds of castor bean (Ricinus communis L.). Cell Biosci 2014, 4:33. 10.1186/2045-3701-4-33 PMC4109380 25061509

[42] Dugas DV, Monaco MK, Olsen A, Klein RR, Kumari S, Ware D, Klein PE (2011). Functional annotation of the transcriptome of Sorghum bicolor in response to osmotic stress and abscisic acid. BMC Genomics.

[43] Li J, Wu Z, Cui L, Zhang T, Guo Q, Xu J (2014). Transcriptome comparison of global distinctive features between pollination and parthenocarpic fruit set reveals transcriptional phytohormone cross-talk in cucumber (Cucumis sativus L.). Plant Cell Physiol.

[44] Sakai A, Yashiro K, Kawano S, Kuroiwa T (1996). Amyloplast formation in cultured tobacco cells; effects of plant hormones on multiplication, size, and starch content. Plant Cell Rep.

[45] Fletcher RA, McCullag D (1971). Cytokinin-induced chlorophyll formation in cucumber cotyledons. Planta.

[46] Yang J, Peng S, Visperas RM, Sanico AL, Zhu Q, Gu S (2000). Grain filling pattern and cytokinin content in the grains and roots of rice plants. Plant Growth Regul.

[47] To JPC, Kieber JJ (2008). Cytokinin signaling: two-components and more. Trends Plant Sci.

[48] Werner T, Schmuelling T (2009). Cytokinin action in plant development. Curr Opin Plant Biol.

[49] Pilkington SM, Montefiori M, Galer AL, Emery RJN, Allan AC, Jameson PE (2013). Endogenous cytokinin in developing kiwifruit is implicated in maintaining fruit flesh chlorophyll levels. Ann Bot.

[50] Akihiro T, Mizuno K, Fujimura T (2005). Gene expression of ADP-glucose pyrophosphorylase and starch contents in rice cultured cells are cooperatively regulated by sucrose and ABA. Plant Cell Physiol.

[51] Gomez-Cadenas A, Zentella R, Walker-Simmons MK, Ho THD (2001). Gibberellin/abscisic acid antagonism in barley aleurone cells: Site of action of the protein kinase PKABA1 in relation to gibberellin signaling molecules. Plant Cell.

[52] Hubbard KE, Nishimura N, Hitomi K, Getzoff ED, Schroeder JI (2010). Early abscisic acid signal transduction mechanisms: newly discovered components and newly emerging questions. Gene Dev.

[53] Park S-Y, Fung P, Nishimura N, Jensen DR, Fujii H, Zhao Y (2009). Abscisic acid inhibits type 2C protein phosphatases via the PYR/PYL family of START proteins. Science.

[54] Umezawa T, Nakashima K, Miyakawa T, Kuromori T, Tanokura M, Shinozaki K (2010). Molecular basis of the core regulatory network in ABA responses: sensing. Signaling Trans Plant Cell Physiol.

[55] Rook F, Corke F, Card R, Munz G, Smith C, Bevan MW (2001). Impaired sucrose-induction mutants reveal the modulation of sugar-induced starch biosynthetic gene expression by abscisic acid signalling. Plant J.

[56] Mares DJ, Marschner H, Krauss A (1981). Effect of gibberellic-acid on growth and carbohydrate-metabolism of developing tubers of potato (solanum-tuberosum). Physiol Plant.

[57] Kaur S, Gupta AK, Kaur N (1998). Gibberellin A3 reverses the effect of salt stress in chickpea (Cicer arietinum L.) seedlings by enhancing amylase activity and mobilization of starch in cotyledons. Plant Growth Regul.

[58] Rentzsch S, Podzimska D, Voegele A, Imbeck M, Mueller K, Linkies A (2012). Dose- and tissue-specific interaction of monoterpenes with the gibberellin-mediated release of potato tuber bud dormancy, sprout growth and induction of alpha-amylases and beta-amylases. Planta.

[59] Yang J, Zhang J, Wang Z, Zhu Q, Wang W (2001). Hormonal changes in the grains of rice subjected to water stress during grain filling. Plant Physiol.

[60] Smith AM, Zeeman SC, Smith SM (2005). Starch degradation. Annu Rev Plant Biol..

[61] Ghiena C, Schulz M, Schnabl H (1993). Starch degradation and distribution of the starch-degrading enzymes in Vicia faba leaves. Plant Physiol.

[62] Barrett JE, Nell TA (1982). Irrigation interval and growth retardants affect poinsettia development. Proceed Florida State Horticultural Soc..

[63] Davis TD, Steffens GL, Sankhla N (1988). Triazole plant growth regulators. Hortic Rev.

[64] Hoagland DR, Arnon DI: The water-culture method for growing plants without soil. Circular California Agricultural Experiment Station,1950;347:2nd edit pp.32 pp.

[65] Zhang L, Zhao H, Gan M, Jin Y, Gao X, Chen Q (2011). Application of simultaneous saccharification and fermentation (SSF) from viscosity reducing of raw sweet potato for bioethanol production at laboratory, pilot and industrial scales. Bioresour Technol.

[66] Wu Y, Messing J (2010). RNA interference-mediated change in protein body morphology and seed opacity through loss of different Zein Proteins1 C W OA. Plant Physiol.

[67] Ji X, Gai Y, Zheng C, Mu Z (2009). Comparative proteomic analysis provides new insights into mulberry dwarf responses in mulberry (Morus alba L.). Proteomics..

[68] Tárrago JF, Nicolás G (1976). Starch degradation in the cotyledons of germinating lentils. Plant Physiol.

[69] Nakamura Y, Yuki K, Park S-Y, Ohya T (1989). Carbohydrate metabolism in the developing endosperm of rice grains. Plant Cell Physiol.

[70] Grabherr MG, Haas BJ, Yassour M, Levin JZ, Thompson DA, Amit I (2011). Full-length transcriptome assembly from RNA-Seq data without a reference genome. Nat Biotechnol.

[71] Langmead B, Trapnell C, Pop M, Salzberg SL (2009). Ultrafast and memory-efficient alignment of short DNA sequences to the human genome. Genome Biol.

[72] Conesa A, Götz S (2008). Blast2GO: A comprehensive suite for functional analysis in plant genomics. Int J Plant Genomics.

[73] Conesa A, Götz S, García-Gómez JM, Terol J, Talón M, Robles M (2005). Blast2GO: a universal tool for annotation, visualization and analysis in functional genomics research. Bioinformatics.

[74] Oliveros JC (2007). VENNY: An interactive tool for comparing lists with Venn Diagrams.

